# Mucolytic Agents and Statins Use is Associated with a Lower Risk of Acute Exacerbations in Patients with Bronchiectasis-Chronic Obstructive Pulmonary Disease Overlap

**DOI:** 10.3390/jcm7120517

**Published:** 2018-12-04

**Authors:** Vincent Yi-Fong Su, Diahn-Warng Perng, Ting-Chun Chou, Yueh-Ching Chou, Yuh-Lih Chang, Chia-Chen Hsu, Chia-Lin Chou, Hsin-Chen Lee, Tzeng-Ji Chen, Po-Wei Hu

**Affiliations:** 1 Department of Internal Medicine, Taipei City Hospital Yangming Branch, Taipei 11146, Taiwan; bsbipoke@hotmail.com; 2 Institute of Clinical Medicine, National Yang-Ming University, Taipei 11221, Taiwan; 3 Faculty of Medicine, School of Medicine, National Yang-Ming University, Taipei 11221, Taiwan; dwperng@vghtpe.gov.tw (D.-W.P.); tjchen@vghtpe.gov.tw (T.-J.C.); 4 Department of Chest Medicine, Taipei Veterans General Hospital, Taipei 11217, Taiwan; peterhu824@yahoo.com.tw; 5 Department of Pharmacy, Taipei Veterans General Hospital, Taipei 11217, Taiwan; tina814a@livemail.tw (T.-C.C.); cchsu6@vghtpe.gov.tw (C.-C.H.); clchou6@vghtpe.gov.tw (C.-L.C.); 6 Department and Institute of Pharmacology, National Yang-Ming University, Taipei 11221, Taiwan; hclee2@ym.edu.tw; 7 School of Pharmacy, Taipei Medical University, Taipei 11031, Taiwan; 8 Department of Family Medicine, Taipei Veterans General Hospital, Taipei 11217, Taiwan; 9 Institute of Hospital and Health Care Administration, National Yang-Ming University, Taipei 11221, Taiwan

**Keywords:** bronchiectasis, COPD, acute exacerbation, BCO

## Abstract

Background: Bronchiectasis-chronic obstructive pulmonary disease (COPD) overlap (BCO) is a neglected area of trials, and it is not covered by guidelines for clinical practice. Methods: Using the National Health Insurance Research Database of Taiwan, COPD patients with or without bronchiectasis from 2000 to 2009 were enrolled as the BCO and COPD alone cohorts, respectively. Patients followed for <28 days, diagnosed with COPD who were not prescribed with COPD medications, and those diagnosed with bronchiectasis who did not receive a chest X-ray or computed tomography were excluded. The primary endpoints were acute exacerbations and mortality. Results: There were 831 patients in the BCO cohort and 3321 patients in the COPD alone cohort, covering 3763.08 and 17,348.95 person-years, respectively, from 2000 to 2011. The BCO cohort had higher risk for exacerbations (adjusted hazard ratio (HR) 2.26, 95% confidence interval (CI) 1.94–2.63) and mortality (HR 1.46, 95% CI 1.24–1.73) than the COPD alone cohort. In the patients overall, the use of statins, macrolides, and mucolytic agents was associated with significantly lower risks of acute exacerbations (statins, HR 0.37, 95% CI 0.29–0.46; macrolides, HR 0.65, 95% CI 0.45–0.93; mucolytic agents, HR 0.68, 95% CI 0.59–0.78). Statins were associated with a significantly lower risk of mortality (HR 0.32, 95% CI 0.25–0.41). In the BCO group, statins and mucolytic agents use was associated with significantly lower risks of acute exacerbations (statins, HR 0.44, 95% CI 0.29–0.65; mucolytic agents, HR 0.58, 95% CI 0.45–0.75). Conclusion: Statins and mucolytic agents use may lower risk of acute exacerbation in patients with BCO.

## 1. Introduction

Chronic obstructive pulmonary disease (COPD) is a chronic inflammatory airway disease which results in high morbidity and mortality [1]. COPD is an important public health problem, and experts have reported that it remains a growing, but neglected global epidemic. COPD is currently the third leading cause of death worldwide in 2016 according to the World Health Organization [2]. COPD and bronchiectasis are different diseases but share many characteristics, including physiopathological and clinical presentations [3]. COPD and bronchiectasis can overlap in some patients, the so called “bronchiectasis-COPD overlap” (BCO) [3]. Based on current guidelines for COPD [1], BCO is diagnosed by radiological and clinical features. The importance of BCO has been emphasized in COPD [1] and bronchiectasis [4] guidelines. Importantly, patients with BCO have a higher risk of acute exacerbations [5] and mortality [6,7] than those with COPD alone. The aim of the present study was to evaluate medications effect on acute exacerbations in patients with BCO. A nationwide population-based study was conducted to analyze the effects of mucolytic agents, macrolides, and statins on acute exacerbations in patients with BCO.

## 2. Methods

### 2.1. Data Source

The Taiwan National Health Research Institute (NHRI) managed and released the National Health Insurance Research Database (NHIRD) for research purposes. The National Health Insurance (NHI) program of Taiwan has been operational in Taiwan since 1995 to provide comprehensive medical services for all Taiwanese citizens [8]. In 2010, the NHIRD contains the claims data of 23 million individuals, which provides coverage to >99% of the entire population of Taiwan. The NHI medical claims databases contain information on demographic characteristics, diseases diagnosis, treatment and related medical expenditures, and orders of ambulatory and inpatient care. From NHIRD, all patients diagnosed with COPD and bronchiectasis were selected. This study used claims data from the Longitudinal Health Insurance Database 2000 (LHID2000) and LHID2005, which contains all the original claims data of 1 million beneficiaries randomly sampled from the NHIRD from 1995 to 2011. The released database has been validated by the NHRI to be representative of the total population in Taiwan. The accuracy of diagnoses recorded in the NHIRD, such as sleep apnea [8], pneumonia [8], asthma [9], COPD [9], tuberculosis [10], and tuberculosis contact [11] has been validated. This study was approved by the Institutional Review Board of Taipei Veterans General Hospital (2017-09-002AC). 

### 2.2. Study Design and Population

In this retrospective cohort study, we analyzed the effects of medications on acute exacerbations in patients with BCO. We enrolled adult patients (≥40 years old) who were newly diagnosed with COPD from 2000 to 2009. The inclusion criteria were patients with a diagnosis of COPD with at least two recorded outpatient visits or two emergency room visits or one hospitalization for COPD. Patients with COPD were defined according to the International Classification of Diseases, Ninth Revision, Clinical Modification (ICD-9-CM) codes 491, 492, 496. Patients with a diagnosis of COPD who were not prescribed with COPD medications within 1 year were excluded. Patients with bronchiectasis were identified by the ICD-9-CM code 494. Patients with a diagnosis of bronchiectasis who did not receive a chest X-ray or computed tomography within 180 days prior to the diagnosis were excluded. The overall cohort of patients with newly-diagnosed COPD was then divided into two cohorts: those with COPD without bronchiectasis (COPD alone group), and those with COPD with concurrent bronchiectasis (BCO group). The index date was defined as the first date on which bronchiectasis was initially diagnosed. Four age-, gender-, and year of enrollment-matched groups of patients with COPD without bronchiectasis were randomly selected from the same datasets. Participants were followed until death, withdrawal from the NHI program, or 31 December 2011, whichever occurred first. Data on the use of mucolytic agents, macrolides, and statins were extracted. The occurrence of acute exacerbations of COPD and bronchiectasis, regardless of which occurred first, was analyzed. We also excluded patients with a follow-up period <28 days, and who had received a diagnosis of bronchiectasis before the study period. 

### 2.3. Potential Confounders and Classification of Severity

To investigate the effects of medications on reducing acute exacerbations, we collected and analyzed age, sex, comorbidities, and severity of COPD. In addition, we assessed diabetes mellitus, cardiovascular disease, stroke, chronic kidney disease, antecedent pneumonia, and malignancy as comorbidities.

Based on the international guideline for COPD [1], the frequency of past exacerbations has been recommended as an important factor in classifying the severity of COPD. The severity of COPD was estimated by the annual frequency of hospitalizations (0 and ≥1) and emergency department visits (0 and ≥1) for acute exacerbations and COPD medications (0–2 and ≥3) in the year before the index date. COPD medications included long-acting β2-agonists, long-acting muscarinic antagonists, short-acting β2-agonists, short-acting muscarinic antagonists, inhaled corticosteroids, and xanthine.

### 2.4. Effect of Exposure to Co-Medications

Information on the prescriptions of co-medications during the follow-up period was extracted to analyze the effects on the development of acute exacerbations in the COPD and BCO cohorts. The patients who were prescribed with co-medications for ≥28 days were assigned to the respective co-medication groups. The three groups of COPD co-medications were macrolides (azithromycin, clarithromycin, erythromycin, josamycin, midecamycin, oleandomycin, roxithromycin, spiramycin, telithromycin, and troleandomycin); mucolytic agents (acetylcysteine, carbocysteine, ambroxol, iodinated glycerol, bromhexine, mesna, and eprazinone); and statins (simvastatin, lovastatin, pravastatin, fluvastatin, atorvastatin, rosuvastatin, and pitavastatin). 

### 2.5. Statistical Analysis

All analyses were conducted using the SAS software (Version 9.4; SAS Institute, Cary, NC, USA). The differences in baseline characteristics and comorbidities between the two groups were examined using the independent Student’s *t*-test (for continuous variables) or Pearson’s χ^2^ test (for categorical variables), as appropriate. The Kaplan-Meier method was used to estimate the outcomes. The differences between the curves were assessed by the log-rank test. Cox proportional-hazards regression models were used to determine the effect of the use of COPD co-medications on reducing acute exacerbations. The proportional hazards assumption was fulfilled in this study. Variables with a p value less than 0.1 in the univariate analyses were entered into the multivariate analysis. All data are expressed as means ± standard deviation or numbers and percentages unless otherwise stated. Two-tailed *p* values < 0.05 were considered to be significant. All analyses were conducted using SAS statistical software, version 9.4.

## 3. Results

### 3.1. Clinical Characteristics of the Study Population

A total of 71,185 patients with newly diagnosed COPD between 1 January 2000 and 31 December 2009 were identified. Of these patients, 2906 who received a diagnosis of bronchiectasis before COPD and those with a follow-up period <28 days (*n* = 4826) were excluded. After excluding those with bronchiectasis who did not undergo chest computed tomography or an X-ray within 180 days (*n* = 2848), the BCO cohort consisted of 831 subjects. Another 3321 age-, gender- and index year -matched COPD patients without bronchiectasis were randomly enrolled in the COPD alone cohort. Data for the BCO (COPD + bronchiectasis) and COPD alone cohorts covered 3763.08 and 17,348.95 person-years, respectively, from 2000 to 2011. Supplemental Figure S1 shows a flow chart of the study population selection.

The baseline characteristics of the BCO and COPD alone cohorts are shown in Table 1. The BCO cohort had a shorter follow-up period (4.53 vs. 5.22 years), higher rates of COPD-related emergency room visits (≥1 visit, 9.63% vs. 3.22%) and hospitalizations (≥1 hospitalization, 20.94% vs. 11.38%), and more prescriptions of COPD medications (≥3 drugs, 36.82% vs. 14.78%; all *p* < 0.0001) compared with the COPD alone cohort. In addition, the BCO cohort had higher prevalence rates of stroke (19.98% vs. 15.45%, *p* = 0.0016), antecedent pneumonia (34.78% vs. 9.55%, *p* < 0.0001), and malignancy (21.78% vs. 10.87%, *p* < 0.0001; Table 1). The co-medications prescribed to both cohorts are shown in Table 1. During the entire follow-up period, significantly more prescriptions for macrolides and mucolytic agents but fewer prescriptions for statins were given to the BCO group compared to the COPD alone group (macrolides, 14.20% vs. 4.91%; mucolytic agents, 85.68% vs. 67.90%; statins, 17.45% vs. 25.62%; all *p* < 0.0001). Similarly, significantly more prescriptions for macrolides and mucolytic agents but fewer prescriptions for statins before the first acute exacerbation were given to the BCO group than the COPD alone group (macrolides, 8.42% vs. 3.64%; mucolytic agents, 76.77% vs. 62.96%; statins, 13.60% vs. 24.24%; all *p* < 0.0001).

### 3.2. The Effect of Bronchiectasis on Acute Exacerbations and Mortality

Table 2 shows the exacerbation rates and medications used to treat the exacerbations in both cohorts. Compared with the COPD alone group, the BCO group had significantly more total exacerbations (rate ratio (RR), 3.42; 95% confidence interval (CI), 3.17–3.68, *p* < 0.0001), outpatient department visits (RR, 3.59; 95% CI, 3.28–3.93, *p* < 0.0001), emergency department visits (RR, 2.79; 95% CI, 1.87–4.13, *p* < 0.0001), hospital admissions (RR, 3.14; 95% CI, 2.72–3.61, *p* < 0.0001), intensive care unit admissions (RR, 4.34; 95% CI, 3.05–6.18, *p* < 0.0001), and days of hospitalization (RR, 3.38; 95% CI, 3.27–3.49, *p* < 0.0001). In addition, the BCO group were prescribed with significantly more COPD medications for exacerbations than the COPD alone group (steroids, 281.2 vs. 208.0 mg, *p* < 0.0001; antibiotics, 14.90 vs. 12.63 days, *p* = 0.0011; anti-pseudomonal fluoroquinolones, 15.03% vs. 8.96%, *p* < 0.0001; mucolytic agents, 60.78% vs. 56.75%, *p* = 0.0301). 

**Figure 1:**
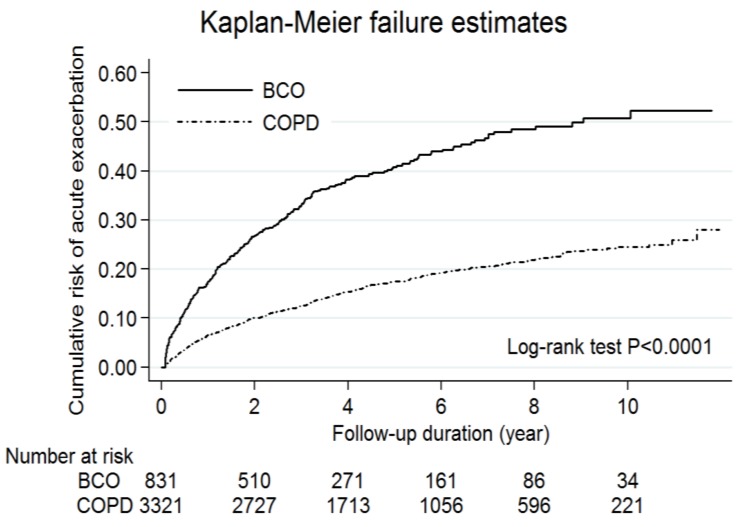
Kaplan-Meier curves for the cumulative risk of acute exacerbations in the patients with chronic obstructive pulmonary disease (COPD) and bronchiectasis-COPD overlap (BCO). There was a statistically significant difference between the two curves (log-rank test, *p* < 0.0001).

**Figure 2:**
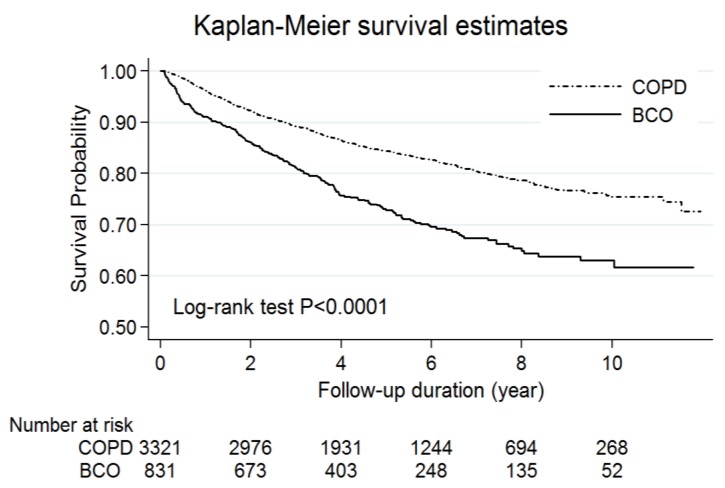
Kaplan-Meier curves for cumulative survival probability in the patients with chronic obstructive pulmonary disease (COPD) and bronchiectasis-COPD overlap (BCO). There was a statistically significant difference between the two curves (log-rank test, *p* < 0.0001).

Of note, the BCO group had a significantly higher risk of acute exacerbations than the COPD alone group in Kaplan-Meier analysis (log-rank test, *p* < 0.0001; Figure 1). After multivariate adjustments, bronchiectasis was independently associated with acute exacerbations (adjusted hazard ratio (HR): 2.26, 95% CI: 1.94–2.63, *p* < 0.0001; Supplemental Table S1). Similarly, the BCO group had a significantly higher risk of mortality than the COPD alone group (log-rank test, *p* < 0.0001; Figure 2). Bronchiectasis was independently associated with mortality (HR: 1.46, 95% CI: 1.24–1.73, *p* < 0.0001; Supplemental Table S2).

### 3.3. Effects of Co-medications on Acute Exacerbations and Mortality

In the COPD patients (the COPD alone and BCO groups), the use of statins, macrolides and mucolytic agents was associated with a significantly lower risk of acute exacerbations (statins, HR 0.37, 95% CI 0.29–0.46, *p* < 0.0001; macrolides, HR 0.65, 95% CI 0.45–0.93, *p* = 0.0169; mucolytic agents, HR 0.68, 95% CI 0.59–0.78, *p <* 0.0001; Supplemental Table S1). In addition, the use of statins was associated with a significantly lower risk of mortality (HR 0.32, 95% CI 0.25–0.41, *p* < 0.0001; Supplemental Table S2). 

In the COPD alone group, the use of statins, macrolides and mucolytic agents was associated with significantly lower risks of acute exacerbations (statins, HR 0.35, 95% CI 0.26–0.46, *p* < 0.0001; macrolides, HR 0.46, 95% CI 0.25–0.83, *p* = 0.0103; mucolytic agents, HR 0.73, 95% CI 0.61–0.86, *p =* 0.0003). In the BCO group, the use of statins and mucolytic agents was associated with significantly lower risks of acute exacerbations (statins, HR 0.44, 95% CI 0.29–0.65; mucolytic agents, HR 0.58, 95% CI 0.45–0.75; all *p <* 0.0001), however the use of macrolides was associated with a non-significantly lower risk of acute exacerbations (macrolides, HR 0.85, 95% CI 0.54–1.32, *p =* 0.4611; Table 3).

### 3.4. Sensitivity and Subgroup Analyses for the Effects of Co-Medications on Acute Exacerbations

Table 3 shows the results of sensitivity analysis for the effects of co-medications on acute exacerbations. In both cohorts, the use of statins and mucolytic agents was associated with a significantly lower dose-dependent risk of acute exacerbations (BCO cohort: (statins, 28–90 days, HR, 0.77; 95% CI, 0.38–1.57, *p* = 0.4647; statins, >90 days, HR, 0.37; 95% CI, 0.23–0.59, *p* < 0.0001) and (mucolytic agents, 28–90 days, HR, 0.80; 95% CI, 0.60–1.08, *p* = 0.1504; mucolytic agents, >90 days, HR, 0.48; 95% CI, 0.36–0.63, *p* < 0.0001)); (COPD alone cohort: (statins, 28–90 days, HR, 0.71; 95% CI, 0.46–1.08, *p* = 0.1071; statins, >90 days, HR, 0.26; 95% CI, 0.18–0.37, *p* < 0.0001) and (mucolytic agents, 28–90 days, HR, 0.82; 95% CI, 0.66–1.01, *p* = 0.0638; mucolytic agents, >90 days, HR, 0.67; 95% CI, 0.55–0.82, *p* < 0.0001)). In contrast, the use of macrolides had no dose–response relationship with the risk of acute exacerbations in either cohort. Supplemental Table S3 shows the percentage of each drug used in both cohorts, and Table 4 shows the results of subgroup analysis for the effect of each drug on acute exacerbations in both cohorts.

## 4. Discussion

In this study, we found health care services were markedly increased in the BCO cohort compared to the COPD alone cohort. Our study showed that the use of statins and mucolytic agents was associated with a decreased risk of acute exacerbation in the patients with BCO. Until now, this is the largest cohort study to investigate the effect of co-medications on the risk of acute exacerbations among patients with BCO.

Because the patients were collected from a national health registry, the selection bias could be minimized. The NHI program provides coverage to almost all residents of Taiwan, thereby minimizing referral bias. The diagnosis of COPD required a prescription with at least one COPD medication, and the diagnosis of bronchiectasis required chest X-ray or computed tomography confirmation. In compliance with the international guideline [1], COPD disease severity could be classified according to the average number of acute exacerbations per year. We used sensitivity analysis to explore the effect of co-medications on acute exacerbations in both cohorts. 

Previous epidemiological studies [5,6,12,13,14] have shown that BCO was associated with more respiratory symptoms and acute exacerbations compared to patients with COPD alone. In this study, the BCO cohort had more medications and acute exacerbations than the COPD alone cohort, which is consistent with previous studies. Two cohort studies [6,12] conducted by Martinez-Garcia et al. in Spain enrolled 92 patients and 201 patients with COPD, respectively, and showed that bronchiectasis was associated with a higher risk of hospitalization due to exacerbations of COPD in the previous year (odds ratio, 3.07; 95% CI, 1.07–8.77) and an independently increased risk of all-cause mortality (HR, 2.54; 95% CI, 1.16–5.56) in patients with COPD. In this study, the BCO group had more total exacerbations (RR, 3.42), outpatient department visits (RR, 3.59), emergency department visits (RR, 2.79), hospital admissions (RR, 3.14), intensive care unit admissions (RR, 4.34), and medications for exacerbations than the COPD alone cohort. After adjustments, bronchiectasis was associated with higher risks of acute exacerbations (HR, 2.26) and mortality (HR, 1.46). 

Mucolytic agents can be a safe, effective, and inexpensive option for the management of COPD and bronchiectasis. However, mucolytic agents do not improve lung function or the risk for death. A previous meta-analysis reported that mucolytic agents were significantly effective in reducing acute COPD exacerbations [15]. However, very high heterogeneity was noted in the meta-analysis, and so the results need to be interpreted with caution. Importantly, mucolytic agents did not show any survival benefits in that meta-analysis. In a more recent systematic review [16], high doses of mucolytic agents had significantly more effect against exacerbations than low doses of mucolytic agents in COPD. Mucus clearance is important in the management of bronchiectasis [17]. Hence, the benefits of mucolytic agents in bronchiectasis remain unclear [18]. High doses of mucolytic agents combined with antibiotics may help with sputum production and clearance, but long-term data and robust clinical outcomes are lacking. One 15-day trial [19] with 88 patients with bronchiectasis showed that high doses of bromhexine eased difficulty in expectoration and reduced sputum production. In this study, we found that mucolytic agents use was associated with a dose-dependent lower risk of acute exacerbations in both the BCO and COPD alone cohorts.

Macrolides have been shown to have immunomodulatory effects and indirect activity against bacterial pathogens through suppression of virulence factors, and this could contribute to the reduction in acute exacerbations of COPD [20,21]. Patients with bronchiectasis have excessive neutrophilic inflammation and a high microbial load in their airways [22]. The ideal treatment for bronchiectasis would break the vicious cycle of mucus stasis, bacterial infection, neutrophilic inflammation, and airway remodeling [23]. Long-term antibiotic therapy might be beneficial for patients with bronchiectasis by reducing bacterial loads. It is not surprising that the duration of antibiotic therapy was significantly longer in the BCO cohort than the COPD alone cohort in this study. In this study, macrolides use was associated with a decreased risk of acute exacerbations in the patients with COPD and a non-significantly decreased risk of acute exacerbations in the patients with BCO. However, only 8.42% of the patients with BCO used macrolides, and the detection power was very low in the BCO cohort.

Statins have been proven to have anti-inflammatory and immunomodulatory effects [24], including reducing proinflammatory cytokines and neutrophil migration [25]. Recently, statins have emerged as a possible therapy for COPD by modifying inflammation. A recent systematic review [26] reported that statins may reduce acute exacerbations and mortality in patients with COPD. In addition, a randomized controlled trial [27] in the UK enrolled 60 patients with bronchiectasis, 30 of whom received high-dose atorvastatin (80 mg) therapy and 30 a placebo. The results showed that six months of statins therapy improved coughing on a quality-of-life scale in patients with bronchiectasis. Post-hoc analysis of that trial showed that the patients treated with statins had fewer exacerbations during treatment. In the current study, statins use was associated with a lower risk of acute exacerbations and mortality in both the BCO and COPD alone groups, which is consistent with previous studies. 

The results of our study suggest that mucolytic agents, macrolides and statins use may effectively reduce acute exacerbations in patients with COPD. Our findings also strengthen the evidence for the benefits of treatment with mucolytic agents, macrolides, and statins in patients with BCO, which could improve the current under-prescription of these drugs in clinical practice. As far as we know, this study is the first to investigate the effect of statins on acute exacerbations in BCO.

There are several limitations to this study. First, some personal information, smoking status, family history, images, and results of pulmonary function tests were not available in the claim database. Second, diagnoses of COPD and bronchiectasis that were recorded by clinicians in the real-world setting may be less accurate than those made in a prospective study design. In our previous work [9], the definition of COPD has been validated. The validation study confirmed the accuracy of definitions of COPD (86.2% sensitivity). Third, we could not assess drug adherence directly. Non-adherence to COPD medications can result in underestimation of the actual effects of medications. Finally, the information of symptom scales of COPD is not available in this study. Functional status is highlighted from the guideline as important for exacerbation rate. 

## 5. Conclusions

This study demonstrated a higher risk of acute exacerbations and mortality in patients with BCO than among those with COPD alone. Mucolytic agents and statins use is associated with a lower risk of future acute exacerbations in the patients with BCO. Further clinical trials are necessary to assess the effects of medications in patients with BCO.

## Figures and Tables

**Table 1:** Characteristics of the bronchiectasis-COPD overlap (BCO) and COPD alone cohorts.

Characteristics	BCO Cohort		COPD Alone Cohort		*p* Value
	*n*	%	*n*	%	
*n*	831		3321		
Age, years (mean ± SD)	68.70 ± 12.14		68.61 ± 12.08		0.8433
Age					0.9822
40–49	62	7.46%	248	7.47%	
50–59	136	16.37%	564	16.98%	
60–69	207	24.91%	834	25.11%	
70–79	254	30.57%	1016	30.59%	
≥80	172	20.70%	659	19.84%	
Sex					0.9741
Male	549	66.06%	2196	66.12%	
Female	282	33.94%	1125	33.88%	
Follow-up, years (mean ± SD) ^§^	4.53 ± 3.00		5.22 ± 2.95		<0.0001
COPD severity ^†^					
COPD-related ED visits, *n* (%)					<0.0001
0	751	90.37%	3214	96.78%	
≥1	80	9.63%	107	3.22%	
COPD-related hospitalizations, *n* (%)					<0.0001
0	657	79.06%	2943	88.62%	
≥1	174	20.94%	378	11.38%	
COPD medications ^‡^, *n* (%)					<0.0001
0–2	525	63.18%	2830	85.22%	
≥3	306	36.82%	491	14.78%	
Comorbidities ^#^					
Diabetes mellitus	152	18.29%	622	18.73%	0.7718
Cardiovascular disease	476	57.28%	1846	55.59%	0.3788
Stroke	166	19.98%	513	15.45%	0.0016
Chronic kidney disease	32	3.85%	119	3.58%	0.7126
Antecedent Pneumonia	289	34.78%	317	9.55%	<0.0001
Malignancy	181	21.78%	361	10.87%	<0.0001
Medications during the follow-up period *					
Statins	145	17.45%	851	25.62%	<0.0001
Macrolides	118	14.20%	163	4.91%	<0.0001
Mucolytic agents	712	85.68%	2255	67.90%	<0.0001
Medications, before the first AE ^$^					
Statins	113	13.60%	805	24.24%	<0.0001
Macrolides	70	8.42%	121	3.64%	<0.0001
Mucolytic agents	638	76.77%	2091	62.96%	<0.0001

^§^ Follow-up period was observed from the index date to death, withdrawal from the NHI program, or until December 31, 2011. ^†^ Proxy indicators of COPD severity were observed in the year before the index date. ^‡^ COPD medications included long-acting β2-agonists, long-acting muscarinic antagonists, short-acting β2-agonists, short-acting muscarinic antagonists, inhaled corticosteroid, and xanthine. ^#^ Comorbidities were observed in the year before the index date. * Medications were observed during the follow-up period. ^$^ Medications were observed from the index date to the first acute exacerbation. SD, standard deviation; BCO, bronchiectasis-COPD overlap; COPD, chronic obstructive pulmonary disease; ED, emergency department; AE, acute exacerbation.

**Table 2:** Rate of COPD exacerbations in the bronchiectasis-COPD overlap (BCO) and COPD alone cohorts.

	BCO Cohort		COPD Alone Cohort		Rate Ratio	*p* Value *
	*n*	Rate ^§^	*n*	Rate ^§^		
Total exacerbations ^†^	1224	32.53	1651	9.52	3.42 (3.17–3.68)	<0.0001
Moderate exacerbations ^†^						
OPD visits	841	22.35	1080	6.23	3.59 (3.28–3.93)	<0.0001
ED visits	43	1.14	71	0.41	2.79 (1.87–4.13)	<0.0001
Severe exacerbations ^†^						
Hospital admissions	340	9.04	500	2.88	3.14 (2.72–3.61)	<0.0001
ICU admissions	65	1.73	69	0.40	4.34 (3.05–6.18)	<0.0001
Hospitalization days	6146	163.32	8395	48.39	3.38 (3.27–3.49)	<0.0001
Drugs for exacerbations, (mean ± SD)						
Steroids ^‡^ (mg)	281.2 ± 342.9		208.0 ± 273.5			<0.0001
Antibiotics (days)	14.90 ± 14.78		12.63 ± 11.92			0.0011
Anti-pseudomonal FQs ^#^, event (%)	15.03%		8.96%			<0.0001
Mucolytic agents, event (%)	60.78%		56.75%			0.0301

PY, person-years; OPD, outpatient department; ED, emergency department; ICU, intensive care unit; FQs, fluoroquinolones; BCO, bronchiectasis-COPD overlap; COPD, chronic obstructive pulmonary disease. ^§^ Exacerbation rate per 100 person-years. ^†^ Exacerbations were observed from the index date to the end of the study. ^‡^ Converted to prednisolone (5 mg/pills) equivalent dose. ^#^ Anti-pseudo FQs included ciprofloxacin and levofloxacin. * Statistical significance was determined using the *t* test for normal distribution, Mann-Whitney *U* test for non-normal distribution, and chi-square test for categorical variables.

**Table 3:** Sensitivity analysis for the effects of medications on acute exacerbations in the bronchiectasis-COPD overlap (BCO) and chronic obstructive pulmonary disease (COPD) alone cohorts.

Variables	BCO Cohort		COPD Alone Cohort	
	HR (95% CI)	*p*	HR (95% CI)	*p*
Medications, before first AE				
Statins (total)	0.44 (0.29–0.65)	<0.0001	0.35 (0.26–0.46)	<0.0001
Statins, 28–90 days	0.77 (0.38–1.57)	0.4647	0.71 (0.46–1.08)	0.1071
Statins, >90 days	0.37 (0.23–0.59)	<0.0001	0.26 (0.18–0.37)	<0.0001
Macrolides (total)	0.85 (0.54–1.32)	0.4611	0.46 (0.25–0.83)	0.0103
Macrolides, 28–90 days	0.94 (0.58–1.52)	0.7947	0.41 (0.21–0.79)	0.0077
Macrolides, >90 days	0.53 (0.17–1.67)	0.2795	0.99 (0.25–3.98)	0.9861
Mucolytic agents (total)	0.58 (0.45–0.75)	<0.0001	0.73 (0.61–0.86)	0.0003
Mucolytic agents, 28–90 days	0.80 (0.60–1.08)	0.1504	0.82 (0.66–1.01)	0.0638
Mucolytic agents, >90 days	0.48 (0.36–0.63)	<0.0001	0.67 (0.55–0.82)	<0.0001

AE, acute exacerbation; BCO, bronchiectasis-COPD overlap; HR, hazard ratio; CI, confidence interval; COPD, chronic obstructive pulmonary disease. All factors (age, sex, proxy indicators of COPD severity, comorbidities, and medications) were included in the Cox multivariate analysis.

**Table 4:** Medication effects on acute exacerbations in the bronchiectasis-COPD overlap (BCO) and chronic obstructive pulmonary disease (COPD) alone cohorts.

Variables	BCO Cohort		COPD Cohort	
	Adjusted HR (95% CI)	*p*	Adjusted HR (95% CI)	*p*
Medications, before first AE				
Statins				
Simvastatin	0.38 (0.19–0.77)	0.0066	0.26 (0.15–0.46)	<0.0001
Lovastatin	0.23 (0.07–0.72)	0.0113	0.43 (0.23–0.78)	0.0053
Pravastatin	0.68 (0.28–1.68)	0.4067	0.58 (0.30–1.13)	0.1078
Fluvastatin	0.57 (0.23–1.39)	0.2144	0.31 (0.15–0.66)	0.0024
Atorvastatin	0.28 (0.15–0.53)	<0.0001	0.34 (0.23–0.52)	<0.0001
Rosuvastatin	0.42 (0.20–0.89)	0.0229	0.25 (0.13–0.46)	<0.0001
Macrolides				
Erythromycin	0.89 (0.45–1.74)	0.7303	0.85 (0.35–2.05)	0.7102
Azithromycin	0.30 (0.04–2.13)	0.2278	N/A	N/A
Clarithromycin	1.03 (0.42–2.50)	0.9564	0.77 (0.32–1.86)	0.5595
Mucolytic agents				
N-acetylcysteine	0.75 (0.58–0.98)	0.0359	0.84 (0.68–0.98)	0.0346
Carbocysteine	0.57 (0.34–0.96)	0.0349	0.79 (0.54–1.14)	0.2033
Ambroxol	0.60 (0.48–0.76)	<0.0001	0.78 (0.66–0.93)	0.0055
Iodinated glycerol	0.72 (0.41–1.26)	0.2522	1.01 (0.68–1.51)	0.9444
Bromhexine	0.60 (0.45–0.80)	0.0004	0.84 (0.68–1.02)	0.0843
Mesna	0.50 (0.07–3.62)	0.4949	0.55 (0.08–3.96)	0.5563
Eprazinone	0.63 (0.41–0.98)	0.0413	0.50 (0.33–0.78)	0.0022

BCO, bronchiectasis-COPD overlap; HR, hazard ratio; CI, confidence interval; COPD, chronic obstructive pulmonary disease; AE, acute exacerbation; N/A, Data were not available due to no events occurring in this group. All factors (age, sex, income level, comorbidities, Charlson Comorbidity Index, urbanization level and medications) in univariate analysis were included in the Cox multivariate analysis.

## Supplementary Materials

The following are available online at http://www.mdpi.com/2077-0383/7/12/517/s1, Figure S1: Flow diagram summarizing the process of enrollment, Table S1: Analysis of risk factors for acute exacerbations in both BCO and COPD alone groups by Cox proportional hazards regression analysis, Table S2: Analysis of risk factors for mortality in both the BCO and COPD alone groups by Cox proportional hazards regression analysis, Table S3: Characteristics of the BCO and COPD alone cohorts.
