# Supplementary material for: Mucolytic Agents and Statins Use is Associated with a Lower Risk of Acute Exacerbations in Patients with Bronchiectasis-Chronic Obstructive Pulmonary Disease Overlap
Source: J Clin Med. 2018 Dec 4;7(12):517. doi: 10.3390/jcm7120517 (PMC6306823; doi:10.3390/jcm7120517)
Supplement: Supplementary file 1 [file jcm-07-00517-s001.pdf]

Supplemental Figure 1. Flow diagram summarizing the process of enrollment.

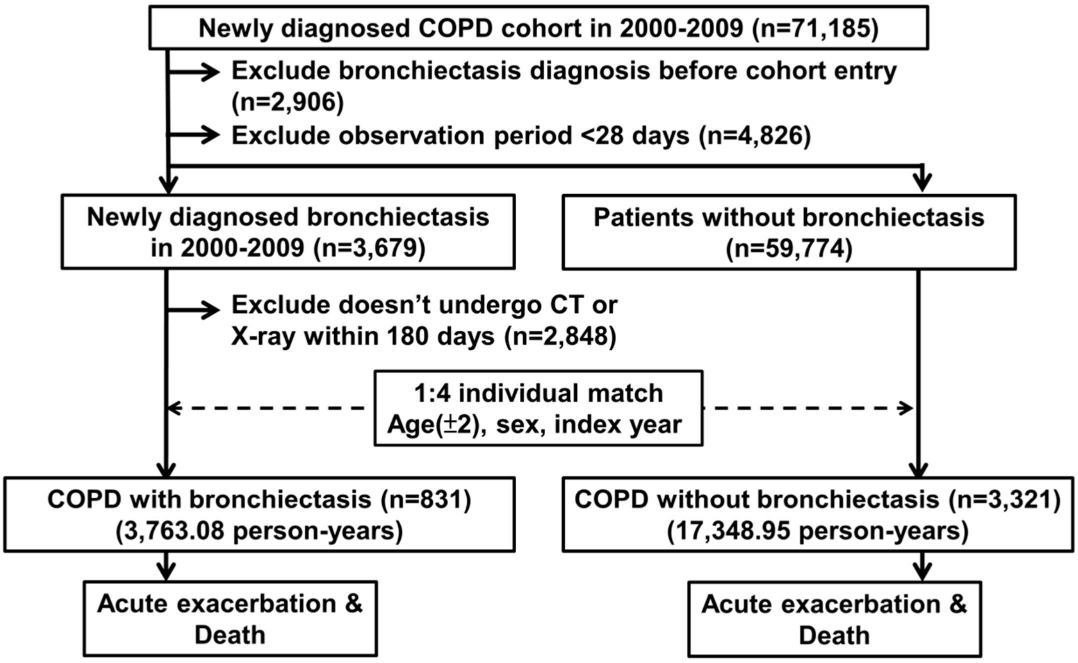

Supplemental Table 1. Analysis of risk factors for acute exacerbations in both BCO and COPD alone groups by Cox proportional hazards regression analysis.

Variables	Unadjusted		Adjusted*	
	HR (95% CI)	*p*	HR (95% CI)	*p*
COPD alone	Reference		Reference	
BCO	2.80 (2.44-3.22)	<.0001	2.26 (1.94-2.63)	<.0001
Age (years)				
40 – 49	Reference		Reference	
50 – 59	1.30 (0.90-1.86)	0.1601	1.24 (0.87-1.78)	0.2407
60 – 69	1.60 (1.14-2.25)	0.0063	1.50 (1.07-2.12)	0.0198
70 – 79	2.59 (1.87-3.59)	<.0001	2.00 (1.44-2.80)	<.0001
≧ 80	3.22 (2.30-4.51)	<.0001	2.20 (1.55-3.11)	<.0001
Sex				
Female	Reference		Reference	
Male	1.66 (1.43-1.94)	<.0001	1.40 (1.18-1.62)	<.0001
COPD severity				
COPD-related ED visits				
0	Reference		Reference	
≥1	3.54 (2.84-4.43)	<.0001	1.63 (1.28-2.07)	<.0001
COPD-related hospitalizations				
0	Reference		Reference	
≥1	2.60 (2.21-3.06)	<.0001	1.25 (1.03-1.50)	0.0208
COPD medications				
0-2	Reference		Reference	
≥3	2.88 (2.50-3.32)	<.0001	1.66 (1.40-1.96)	<.0001
Comorbidities				
Diabetes mellitus	1.17 (0.99-1.39)	0.0661	1.22 (1.02-1.46)	0.0265
Cardiovascular disease	1.34 (1.17-1.54)	<.0001	1.08 (0.93-1.25)	0.3331
Stroke	1.98 (1.69-2.32)	<.0001	1.48 (1.24-1.75)	<.0001
Chronic kidney disease	1.75 (1.26-2.42)	0.0008	1.25 (0.89-1.74)	0.1962
Antecedent Pneumonia	2.96 (2.54-3.45)	<.0001	1.36 (1.14-1.63)	0.0007
Malignancy	1.22 (1.01-1.49)	0.0442	0.85 (0.70-1.05)	0.1250
Medications§, before first AE				
Statins	0.31 (0.25-0.39)	<.0001	0.37 (0.29-0.46)	<.0001
Macrolides	0.76 (0.54-1.09)	0.1343	0.65 (0.45-0.93)	0.0169
Mucolytic agents	0.82 (0.72-0.95)	0.0066	0.68 (0.59-0.78)	<.0001

Abbreviations: SD, standard deviation; BCO, bronchiectasis-COPD overlap; COPD: chronic obstructive pulmonary disease; ED: emergency department; AE, acute exacerbation

§Medications were observed from the index date to the first acute exacerbation, withdrawal from the NHI program, or until December 31, 2011.

*All factors were included in the Cox multivariate analysis.

Supplemental Table 2. Analysis of risk factors for mortality in both the BCO and COPD alone groups by Cox proportional hazards regression analysis.

Variables	Unadjusted		Adjusted*	
	HR (95% CI)	*p*	HR (95% CI)	*p*
COPD alone	Reference		Reference	
BCO	1.81 (1.55-2.12)	<.0001	1.46 (1.24-1.73)	<0.0001
Age (years)				
40 – 49	Reference		Reference	
50 – 59	1.34 (0.77-2.31)	0.2968	1.18 (0.68-2.03)	0.5631
60 – 69	2.11 (1.28-3.48)	0.0035	1.74 (1.05-2.88)	0.0315
70 – 79	5.88 (3.65-9.46)	<.0001	3.97 (2.45-6.45)	<0.0001
≧ 80	12.11 (7.51-19.53)	<.0001	6.78 (4.16-11.04)	<0.0001
Sex				
Female	Reference		Reference	
Male	1.43 (1.22-1.68)	<.0001	1.07 (0.91-1.26)	0.4100
Proxy indicators of COPD severity				
COPD-related ED visits				
0	Reference		Reference	
≥1	2.25 (1.73-2.93)	<.0001	1.02 (0.77-1.35)	0.8893
COPD-related hospitalizations				
0	Reference		Reference	
≥1	3.66 (3.13-4.28)	<.0001	1.78 (1.48-2.14)	<0.0001
COPD medications				
0-2	Reference		Reference	
≥3	2.46 (2.12-2.87)	<.0001	1.28 (1.07-1.53)	0.0064
Comorbidities				
Diabetes mellitus	1.76 (1.50-2.07)	<.0001	1.67 (1.41-1.98)	<.0001
Cardiovascular disease	1.94 (1.67-2.26)	<.0001	1.11 (0.94-1.31)	0.2035
Stroke	2.78 (2.38-3.25)	<.0001	1.62 (1.37-1.91)	<0.0001
Chronic kidney disease	3.55 (2.75-4.58)	<.0001	2.27 (1.75-2.95)	<0.0001
Antecedent Pneumonia	2.92 (2.49-3.42)	<.0001	1.17 (0.98-1.41)	0.0875
Malignancy	2.01 (1.69- 2.40)	<.0001	1.59 (1.32-1.90)	<0.0001
Medications during the follow-up period				
Statins	0.29 (0.23-0.36)	<.0001	0.32 (0.25-0.41)	<0.0001
Macrolides	1.62 (1.29-2.03)	<.0001	1.19 (0.94-1.51)	0.1418
Mucolytic agents	1.34 (1.14-1.58)	0.0003	1.08 (0.90-1.30)	0.3974

Abbreviations: SD, standard deviation; BCO, bronchiectasis-COPD overlap; COPD: chronic obstructive pulmonary disease; ED: emergency department

§ Medications were observed from the index date to death, withdrawal from the NHI program, or until December 31, 2011.

*All factors in univariate analysis were included in the Cox multivariate analysis.

Supplemental Table 3. Characteristics of the BCO and COPD alone cohorts.

**Characteristics**	**BCO cohort**		**COPD alone cohort**		***p* value**
	n	**%**	***n***	**%**	
Medications*, before first AE					
Statins	113	13.60%	805	24.24%	<.0001
Simvastatin	38	4.57%	251	7.56%	
Lovastatin	23	2.77%	150	4.52%	
Pravastatin	14	1.68%	107	3.22%	
Fluvastatin	19	2.29%	118	3.55%	
Atorvastatin	59	7.10%	398	11.98%	
Rosuvastatin	34	4.09%	228	6.87%	
Macrolides	70	8.42%	121	3.64%	<.0001
Erythromycin	25	3.01%	32	0.96%	
Azithromycin	7	0.84%	7	0.21%	
Clarithromycin	19	2.29%	35	1.05%	
Other	0	0.00%	2	0.06%	
Mix	19	2.29%	45	1.36%	
Mucolytic agents	638	76.77%	2091	62.96%	<.0001
N-acetylcysteine	247	29.72%	665	20.02%	
Carbocysteine	63	7.58%	191	5.75%	
Ambroxol	403	48.50%	1237	37.25%	
Iodinated glycerol	41	4.93%	108	3.25%	
Bromhexine	211	25.39%	691	20.81%	
Mesna	4	0.48%	13	0.39%	
Eprazinone	70	8.42%	252	7.59%	

* Medications were observed from the index date to the first acute exacerbation.

Abbreviations: BCO, bronchiectasis-COPD overlap; COPD: chronic obstructive pulmonary disease; AE, acute exacerbation
